# No Glymphatic Dysfunction in Trigeminal Neuralgia: A Preliminary Study

**DOI:** 10.1002/brb3.70938

**Published:** 2026-01-28

**Authors:** Barış Genç, Kerim Aslan

**Affiliations:** ^1^ Faculty of Medicine, Department of Radiology Ondokuz Mayıs University Samsun Türkiye

## Abstract

**Background:**

Trigeminal neuralgia (TN) patients are at risk for glymphatic dysfunction due to their prolonged headaches and sleep disturbances.

**Objective:**

The aim of this study is to compare the glymphatic function changes in TN patients with healthy controls, to investigate whether there are differences in glymphatic function after decompression surgery, and to examine their relationship with clinical parameters.

**Methods:**

The data for this hybrid design study were obtained from the OpenNeuro dataset titled “A large‐scale dataset of pre‐ and post‐surgical MRI data in patients with chronic trigeminal neuralgia.” The DTI‐ALPS index values were calculated for 62 TN patients with normal brain MRIs and 35 age‐ and sex‐matched healthy controls, as well as for 53 TN patients who had both preoperative and postoperative diffusion tensor imaging. Statistical analyses involved nonparametric tests and Spearman correlation to assess differences between groups and relationships with clinical variables such as age, disease duration, and Sindou grading.

**Results:**

There were no significant differences in DTI‐ALPS indices between TN patients and controls. Additionally, preoperative and postoperative comparisons in the TN cohort revealed no significant changes in glymphatic function following surgery. Furthermore, no correlations were observed between DTI‐ALPS indices and clinical parameters, including patient age, disease duration, or the severity of neurovascular compression.

**Conclusions:**

The findings of this preliminary study suggest that glymphatic dysfunction is not a contributing factor in the pathogenesis of TN and that microvascular decompression surgery does not alter glymphatic clearance. These results indicate that the underlying mechanisms of TN may be independent of glymphatic impairment, despite the presence of sleep disturbances in this population.

## Graphical abstract

In this study investigating cross‐sectional and longitudinal DTI‐ALPS changes in patients with trigeminal neuralgia, no DTI‐ALPS alterations were detected in trigeminal neuralgia patients.

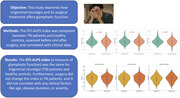

## 1 Introduction

Trigeminal neuralgia is characterized by sudden, transient, and recurrent electric shock–like facial pain. It is the most common form of facial neuropathic pain syndrome, with an incidence of approximately four to five cases per 100,000 individuals (Svedung Wettervik et al. 2023). The most frequent etiology of trigeminal neuralgia involves neurovascular compression at the root entry zone, typically referred to as idiopathic or classical trigeminal neuralgia (Gambeta et al. 2020). This vascular contact and compression lead to demyelination at the site of contact. In classical trigeminal neuralgia, the primary treatment approach consists of microvascular decompression, which targets removal of the underlying etiological factor (Gambeta et al. 2020). In particular, changes in CBF have been observed during trigeminal ganglion stimulation, supporting the hypothesis of trigeminal control over cerebral hemodynamics (Visocchi et al. 1996).

In recent years, the glymphatic system has been recognized as a lymphatic pathway in the brain. It facilitates the clearance of proteins such as lactic acid and amyloid tau from perivascular spaces toward the arachnoid granulations and meningeal lymphatics (Jessen et al. 2015). Dysfunction of this system has been implicated in various neurodegenerative diseases, including Alzheimer's and Parkinson's (Zhou et al. 2024; Kamagata et al. 2022). Moreover, the glymphatic system contributes to the removal of proinflammatory cytokines alongside these neurodegenerative proteins (Szlufik et al. 2024). Many methods have been described for measuring the functions of the glymphatic system, but an optimal method has yet to be established (Kamagata et al. 2024). Diffusion Tensor Image Analysis Along the Perivascular Space (DTI‐ALPS) method is a highly reproducible technique that assesses glymphatic system function by measuring the amount of diffusion in the perivascular spaces (Taoka et al. 2024).

Research has increasingly focused on the potential role of glymphatic dysfunction not only in neurodegenerative disorders but also in headache syndromes (Vittorini et al. 2024). Identifying a link between glymphatic dysfunction and headaches could pave the way for targeted treatments, potentially enhancing therapeutic outcomes (Gao et al. 2023). However, some migraine studies have shown no significant changes in glymphatic function among migraine patients, with the exception of those with chronic migraine, where glymphatic activity was found to be increased and was associated with elevated calcitonin gene‐related peptide (CGRP) levels (D. A. Lee, Lee, and Park 2022; Cha et al. 2024; Burgos et al. 2024; Wu et al. 2024). By contrast, reduced glymphatic function has been detected in patients with intracranial hypertension, suggesting that impaired glymphatic drainage might result in cerebrospinal fluid (CSF) accumulation within nerve sheaths and potentially contribute to pain (Eide et al. 2021). Notably, to date, no study has investigated glymphatic function in patients with trigeminal neuralgia.

Our hypothesis posits that trigeminal neuralgia is associated with glymphatic dysfunction. Several mechanisms may underlie this dysfunction. The glymphatic system is most active during sleep, and patients with trigeminal neuralgia often experience disturbed sleep (Yi et al. 2022). Because the bulk of glymphatic drainage occurs during sleep, sleep disruptions could impair glymphatic clearance. Recent studies have also identified elevated levels of proinflammatory cytokines in the CSF of patients with trigeminal neuralgia, and these cytokines are partly cleared through glymphatic pathways (Lai et al. 2025; Ostertag et al. 2023). Consequently, compromised glymphatic drainage in trigeminal neuralgia may contribute to increased proinflammatory cytokine concentrations. Furthermore, glymphatic dysfunction could potentially create a neurodegenerative substrate in these patients (Szlufik et al. 2024). Indeed, volumetric reductions in supratentorial gray matter and widespread microstructural changes in white matter have been reported in trigeminal neuralgia, with these changes correlating with disease duration (Li et al. 2017; Tan et al. 2022; Ge et al. 2023). To our knowledge, no study thus far has specifically evaluated glymphatic function in this patient population, yet it is plausible that such dysfunction could predispose them to neurodegenerative processes.

The primary aim of our study is to elucidate potential alterations in glymphatic function among individuals with trigeminal neuralgia and to determine the relationship of these alterations with relevant clinical parameters. A secondary objective is to investigate whether glymphatic function is modified following microvascular decompression surgery in this patient cohort.

## 2 Materials and Methods

### 2.1 Study Population

This study has a hybrid design: it is case‐control in nature when comparing the preoperative DTI‐ALPS findings of trigeminal neuralgia patients with those of healthy volunteers, and cohort in nature when evaluating the longitudinal changes by comparing the preoperative and postoperative data within the patient group. Participants in this study were drawn from the OpenNeuro dataset titled “A large‐scale dataset of pre‐ and post‐surgical MRI data in patients with chronic trigeminal neuralgia.” (https://openneuro.org/datasets/ds005713/versions/1.0.1) (de Carvalho Fonseca et al. 2012) The dataset includes 110 patients diagnosed with persistent trigeminal neuralgia and 48 healthy volunteers. Among the patient cohort, 58 individuals underwent follow‐up MRI at 6 months post‐surgery. The dataset also provides clinical parameters, including Sindou grade (reflecting the degree of neurovascular compression), duration of disease, and patient age.

**FIGURE 1:**
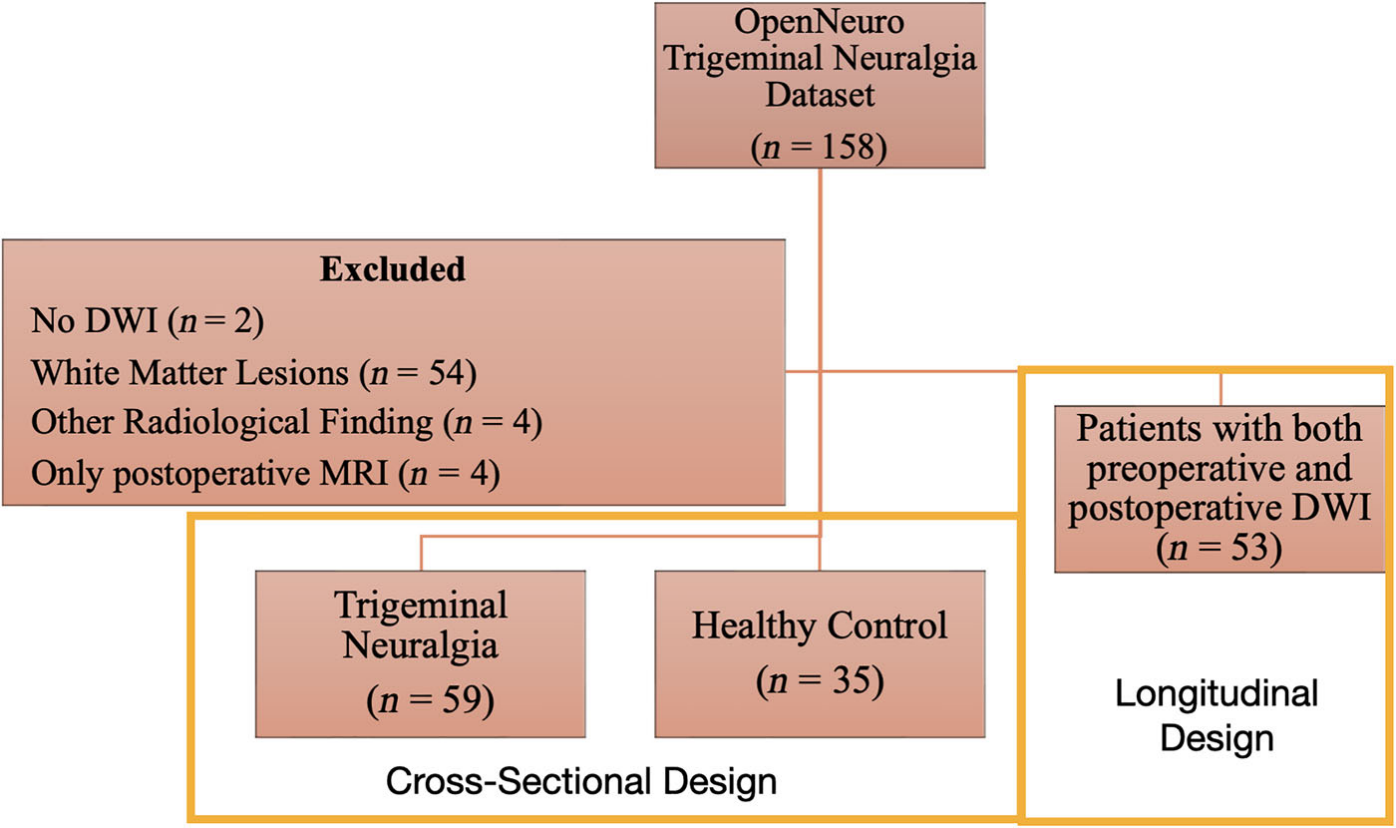
Flowchart of study.

Out of the preoperative patient scans, two (sub‐107 and sub‐128) had only structural MRI (no DTI data), while four postoperative scans (sub‐009fu, sub‐028fu, sub‐068fu, and sub‐087fu) were similarly limited to structural imaging; these individuals were therefore excluded. Additionally, because at least a Fazekas Type‐1 white matter lesion was identified in 11 control participants and 43 patients, those individuals were excluded, as were one patient with a sequela gliotic lesion (sub‐084), one patient with sinus venous thrombosis (sub‐097), one healthy control with a multi‐nodular vacuolar glioneuronal tumor (sub‐45), and one control participant displaying clinical and radiological findings of intracranial hypertension (sub‐23). Among the remaining 110 patients, 53 had usable 6‐month follow‐up DTI scans, enabling comparison of preoperative and postoperative DTI‐ALPS data (Figure 1). The study was designed and reported in accordance with STROBE guidelines (Ghaferi et al. 2021). It has been stated that a large language model was used for incorporating the anime figure into the graphical abstract, as well as for English translation and proofreading.

### 2.2 MRI Acquisition and Preprocessing

**FIGURE 2:**
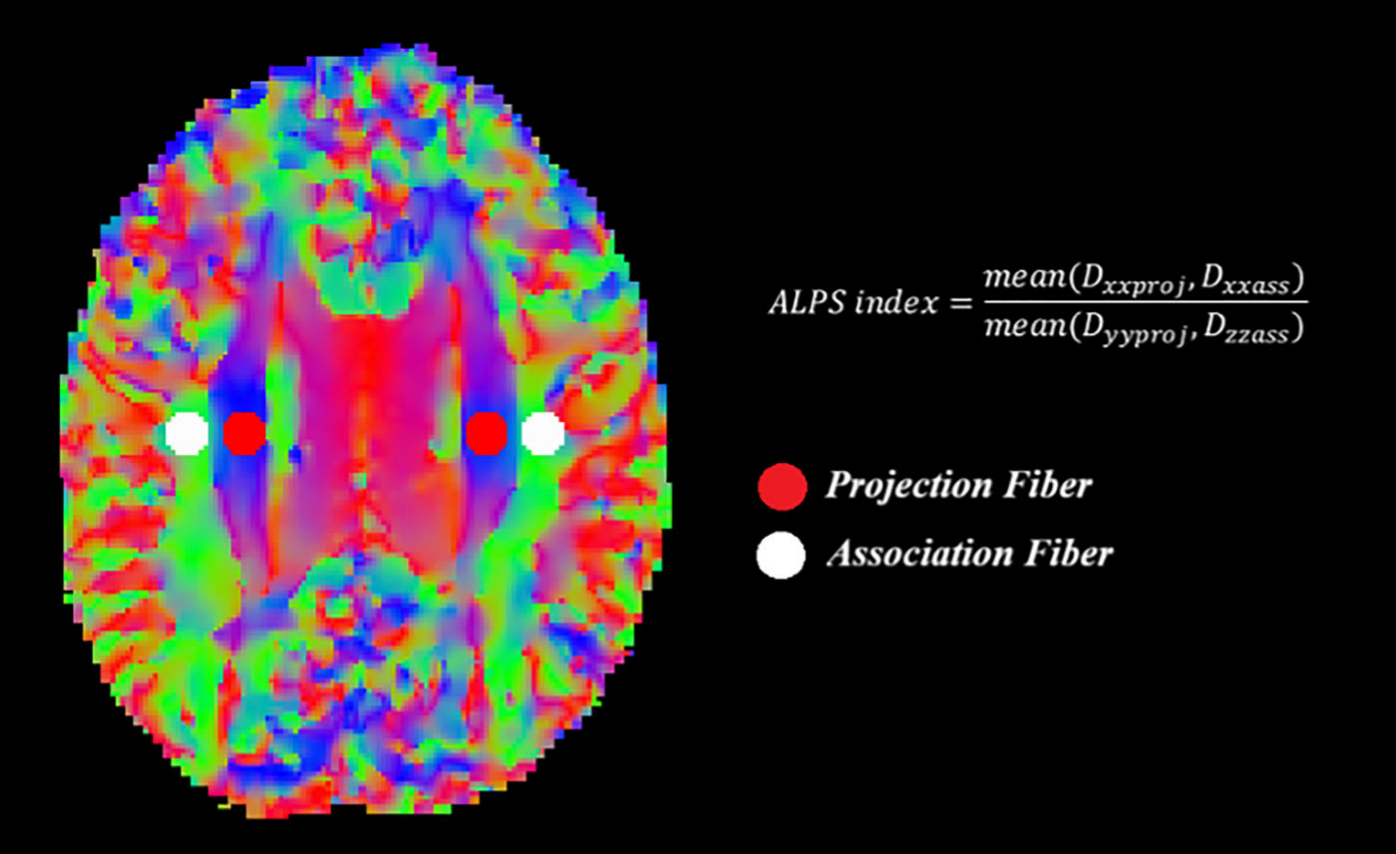
Calculation of the diffusion tensor imaging analysis along the perivascular space (DTI‐ALPS) technique.

All MRI scans were acquired using a Philips Ingenia 3T scanner. Diffusion tensor images were obtained using three‐dimensional echo‐planar imaging (EPI) sequences with a slice thickness of 2 mm. The data included *b*‐values of 1500 s/mm^2^ with 64 diffusion directions, along with b0 images acquired in both anterior‐posterior and posterior‐anterior phase encoding directions (two each). Image preprocessing was performed using MrTrix version 3.0.2 and FSL version 6.0.5. First, noise reduction was carried out using the dwidenoise function, which applies the Marchenko‐Pastur Principal Component Analysis (MP‐PCA) method (Veraart et al. 2016). Next, Gibbs ringing artifacts were minimized using mrdegibbs (Kellner et al. 2016). Subsequently, eddy current and motion artifacts were corrected using dwifslpreproc (Andersson and Sotiropoulos 2016; Skare and Bammer 2010). During this step, susceptibility‐induced distortions were also corrected using the topup tool with the reverse phase‐encoded b0 images. Bias field correction was then performed using the dwibiascorrect command with the ANTs backend. Diffusion tensors were calculated using the dti_fit function in FSL. For DTI‐ALPS analysis, fractional anisotropy (FA) images were initially registered to the JHU atlas using FSL‐FLIRT, and the resulting transformation matrix was applied to the diffusion tensors using “vecreg” to ensure proper alignment. In defining the ROIs for ALPS analysis, projection fiber regions at the level of the lateral ventricle body were identified as the superior corona radiata (SCR), while association fiber regions were identified as the superior longitudinal fasciculus (SLF), in accordance with the JHU‐ICBM‐DTI‐81 white matter atlas. On the JHU template, spherical ROIs with a diameter of 5 mm were automatically positioned bilaterally in the SCR and SLF, with center coordinates as follows: left SCR, left SLF, right SCR, and right SLF. These ROIs were transferred to each participant's diffusivity maps, from which mean diffusivity values along the *x*‐, *y*‐, and *z*‐axes (*D*_xx_, *D*_yy_, and *D*_zz_) were obtained. The ALPS index in each hemisphere was computed by dividing the mean *x*‐axis diffusivity of the projection and association fiber ROIs (*D*_xxproj_ and *D*_xxassoc_) by the mean *y*‐axis diffusivity in the projection fiber ROI (*D*_yyproj_) and the mean z‐axis diffusivity in the association fiber ROI (*D*_zzassoc_). The ALPS index for each hemisphere was obtained by first measuring diffusivity along the *x*‐axis within both the projection fiber ROI and the association fiber ROI. These values were then compared to the diffusivity measured along the *y*‐axis in the projection fiber ROI and along the *z*‐axis in the association fiber ROI. The ratio between the average *x*‐axis diffusivity and the average of the *y*‐ and *z*‐axis diffusivities was taken as the ALPS index. The final ALPS index for each subject was calculated by averaging the values from the left and right hemispheres. An overall mean ALPS index for each subject was then derived by averaging the left and right hemisphere values. Subsequently, the DTI‐ALPS values for both hemispheres were automatically calculated according to the predefined pipeline (Figure 2) (Liu et al. 2024).

### 2.3 Statistical Analysis

Descriptive statistics for the patient and control groups were presented as mean and standard deviation. Differences in gender distribution were assessed using the chi‐square test. Since age and DTI‐ALPS metrics in both groups did not show normal distribution according to the Shapiro–Wilk test, the Mann–Whitney U test was used for group comparisons. Preoperative and postoperative DTI‐ALPS indices in patients were compared using the Wilcoxon signed‐rank test. The correlation between preoperative DTI‐ALPS index values and clinical findings was examined using Spearman correlation analysis. Statistical analyses were conducted using SPSS version 26.0 and Python libraries, including Pandas, SciPy, and Matplotlib. A *p*‐value of < 0.05 was considered statistically significant.

## 3 Results

### 3.1 Clinical and Demographic Data

The mean age was 56.91 ± 7.61 years in the control group and 54.65 ± 12.27 years in the patient group, with no statistically significant difference between the groups (*p* = 0.622). The control group consisted of 19 females and 16 males, while the patient group included 34 females and 25 males. Chi‐square analysis revealed no significant difference in gender distribution between the two groups (*p* = 0.920). Among the patients, 56 were diagnosed with paroxysmal trigeminal neuralgia and six with persistent trigeminal neuralgia. The mean disease duration was 7.89 ± 5.79 years. Regarding the degree of neurovascular conflict based on Sindou grading, eight patients had grade 1, 17 had grade 2, and 14 had grade 3 compression. Symptoms were left‐sided in 29 patients and right‐sided in 33 patients. It was determined that no neurovascular conflict was present in 20 patients.

### 3.2 DTI‐ALPS Findings

**FIGURE 3:**
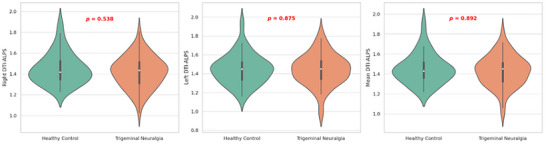
Violin plot showing the DTI‐ALPS index values of the trigeminal neuralgia and control groups.

The right DTI‐ALPS index was 1.45 ± 0.15 in healthy controls and 1.41 ± 0.15 in the patient group, with no statistically significant difference (*p* = 0.538). The left DTI‐ALPS index was 1.44 ± 0.15 in controls and 1.44 ± 0.16 in patients, also showing no significant difference (*p* = 0.875). The mean DTI‐ALPS index was 1.45 ± 0.15 in controls and 1.43 ± 0.15 in the patient group, with no statistically significant difference (*p* = 0.892) (Figure 3) (Table 1).

**TABLE 1:** Clinical and demographic finding and DTI‐ALPS results of patient with trigeminal neuralgia and healthy control.

	Healthy control (*n* = 35)	Trigeminal neuralgia (*n* = 62)	*p* value
Age	56.91 ± 7.61	54.65 ± 12.27	0.622
Female (%)	54%	58%	0.718
Right DTI‐ALPS	1.45 ± 0.15	1.41 ± 0.15	0.538
Left DTI‐ALPS	1.44 ± 0.15	1.44 ± 0.16	0.875
Mean DTI‐ALPS	1.45 ± 0.15	1.43 ± 0.15	0.892

### 3.3 Longitudinal DTI‐ALPS Results

**FIGURE 4:**
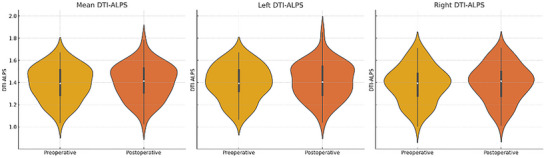
Violin plot showing the DTI‐ALPS index values of the preoperative and postoperative trigeminal neuralgia patients.

In the longitudinal analysis of 53 patients, the right DTI‐ALPS index was 1.39 ± 0.15 preoperatively and 1.39 ± 0.15 postoperatively, with no significant difference (*p* = 0.785). The left DTI‐ALPS index was 1.40 ± 0.16 preoperatively and 1.41 ± 0.16 postoperatively, with no significant difference (*p* = 0.418). The mean DTI‐ALPS index was 1.39 ± 0.15 preoperatively and 1.40 ± 0.15 postoperatively, again showing no statistically significant change (*p* = 0.348) (Figure 4) (Table 2).

**TABLE 2:** Preopertive and postoperative DTI‐ALPS results of trigeminal neuralgia patients.

	Preoperative (*n* = 53)	Postoperative (*n* = 53)	*p* value
Right DTI‐ALPS	1.39 ± 0.15	1.39 ± 0.15	0.785
Left DTI‐ALPS	1.40 ± 0.16	1.41 ± 0.16	0.418
Mean DTI‐ALPS	1.39 ± 0.15	1.40 ± 0.15	0.348

### 3.4 Correlation of DTI‐ALPS Index With Clinical Variables

The mean DTI‐ALPS index showed no statistically significant correlation with patient age (rho = −0.182, *p* = 0.158), disease duration (rho = 0.200, *p* = 0.123), or Sindou grade (rho = −0.173, *p* = 0.179).

## 4 Discussion

The main finding of this study is the absence of evidence for glymphatic dysfunction in patients with trigeminal neuralgia. No association was found between glymphatic function and clinical parameters in these patients. Another key result is that surgical intervention for trigeminal neuralgia did not lead to detectable changes in glymphatic function.

The glymphatic system is a relatively newly discovered drainage pathway. Glymphatic dysfunction has been reported in various neurodegenerative diseases such as Alzheimer's and Parkinson's disease, as well as in inflammatory disorders like Multiple Sclerosis (Zhou et al. 2024; Kamagata et al. 2022; Szlufik et al. 2024; Gao et al. 2023). However, studies investigating the relationship between headache syndromes and glymphatic function are still scarce (Vittorini et al. 2024). Existing research has shown reduced glymphatic function in cluster headache, while no dysfunction has been observed in patients with migraine‐associated persistent headache (Vittorini et al. 2024; D. A. Lee, Lee, and Park 2022; Cha et al. 2024; Burgos et al. 2024; Kim et al. 2022; Burgos et al. 2024). In a study by Kim et al. (2022) on cluster headache, although the findings were relatively notable, the statistical significance was limited and the sample size was small. These findings suggest that the link between headache and glymphatic function may be weaker than previously assumed. Therefore, it may be concluded that glymphatic dysfunction plays a more prominent role in neurodegenerative conditions and is not significantly involved in headache syndromes such as trigeminal neuralgia.

Previous studies have shown reduced sleep quality and shorter sleep duration in patients with trigeminal neuralgia, indicating a disruption of the sleep–wake cycle (Yi et al. 2022; Mishra et al. 2025). Since glymphatic clearance predominantly occurs during sleep, sleep quality and circadian rhythm are thought to be crucial for effective glymphatic function (Chong et al. 2022; H. J. Lee, Lee, Shin, et al. 2022). Despite this, our study did not detect glymphatic dysfunction in patients with trigeminal neuralgia, who are likely to experience sleep disturbances. Similarly, previous studies on migraine and other headache disorders have not shown glymphatic dysfunction despite reported sleep impairment (Vittorini et al. 2024). For instance, Zhang et al. (2023) found no glymphatic dysfunction in patients with new daily persistent headache (NDPH), even though half of the patients experienced sleep disorders. No correlation was found between sleep parameters and the ALPS index. These results suggest that sleep disturbances observed in conditions such as trigeminal neuralgia, migraine, and NDPH may not have the expected detrimental effect on glymphatic function. This implies that sleep‐dependent glymphatic clearance mechanisms may not be measurably impaired in clinical sleep disturbances. It is possible that either the measurement techniques, such as DTI‐ALPS, are limited in detecting the link between sleep and glymphatic function, or that the glymphatic system may possess compensatory mechanisms that provide resilience against sleep disturbances. In fact, animal studies have shown that sleep‐deprived migraine‐model mice exhibit glymphatic dysfunction (W. Huang et al. 2023). Future studies specifically evaluating the effect of sleep disorders on the glymphatic system will be valuable to better understand this relationship.

Microvascular decompression surgery is currently one of the most effective treatments for trigeminal neuralgia, significantly reducing symptoms in affected patients. Despite symptom relief following surgery, our findings demonstrate that glymphatic function remains unchanged postoperatively. This suggests that glymphatic assessment may not be clinically relevant for the management or follow‐up of patients with trigeminal neuralgia.

The DTI‐ALPS method is a noninvasive, repeatable, easily computable, and workflow‐compatible technique for evaluating glymphatic function (Liu et al. 2024). Correlations between DTI‐ALPS indices and clinical parameters have been reported in neurodegenerative conditions such as Alzheimer's disease, Parkinson's disease, and normal pressure hydrocephalus (S. Huang et al. 2024; Reeves et al. 2020; Zhou et al. 2024). However, there is still no consensus on the optimal method for assessing glymphatic function. This is partly due to the fact that perivascular spaces represent only about 1% of cerebral tissue, making it challenging to isolate their contribution to diffusion measures. Moreover, glymphatic drainage predominantly occurs in the subcortical regions, while the DTI‐ALPS method measures diffusion characteristics in regions of association and projection fibers. This indirect measurement may be prone to error (Ringstad 2024). Additionally, factors such as head positioning and motion can affect DTI‐ALPS measurements. In this study, we applied several correction algorithms and utilized the “vecreg” function within FSL to address these issues, as recommended in the literature (Tatekawa et al. 2023). Nevertheless, our initial results indicate no evidence of glymphatic dysfunction in patients with trigeminal neuralgia.

To the best of our knowledge, this is the first study to investigate glymphatic function in patients with trigeminal neuralgia. We carefully ensured that both the patient and control groups had normal brain MRIs aside from neurovascular conflict, thereby minimizing the potential confounding effect of small vessel disease. A homogeneous population was selected, and DTI‐ALPS indices were evaluated using standardized measurements both preoperatively and postoperatively. However, our study has several limitations. First, as discussed earlier, the DTI‐ALPS method has inherent limitations in assessing glymphatic function. Second, the MRI data used in this study were obtained from the OpenNeuro repository, limiting the available clinical parameters. For example, data regarding sleep duration or sleep quality were not available. Additionally, MRI scans were acquired while subjects were awake, whereas glymphatic drainage occurs predominantly during sleep. This may have hindered the detection of potential sleep‐related glymphatic dysfunction. Future studies designed to overcome these limitations may provide a more accurate assessment of glymphatic function in trigeminal neuralgia.

In conclusion, these preliminary results demonstrate that glymphatic function is not impaired in patients with trigeminal neuralgia. Surgical treatment did not lead to changes in glymphatic function. No association was found between DTI‐ALPS indices and clinical variables such as disease duration or degree of neurovascular conflict. Future studies are warranted to further explore and validate these findings.

## Author Contributions

**Barış Genç**: Project development, Data collection, Data analysis, Manuscript writing/editing. **Kerim Aslan**: Manuscript editing.

## Data Availability

The data that support the findings of this study are available in A large‐scale dataset of pre‐ and post‐surgical MRI data in at https://openneuro.org/datasets/ds005713/versions/1.0.1, reference number 22. These data were derived from the following resources available in the public domain:—doi:10.18112/openneuro.ds005713.v1.0.1, https://openneuro.org/datasets/ds005713/versions/1.0.1.
